# Gradual Treatment of Arteriovenous Fistula in Femoral Vessels as a Complication of Coronary Angiography

**DOI:** 10.21470/1678-9741-2018-0008

**Published:** 2018

**Authors:** Mehmet Işık, Ömer Tanyeli, Yüksel Dereli, Volkan Burak Taban, Özgür Altınbaş, Niyazi Görmüş

**Affiliations:** 1 Department of Cardiovascular Surgery, Necmettin Erbakan University, Meram Medicine Faculty, Konya, Turkey.; 2 Department of Cardiovascular Surgery, Training and Research Hospital, Konya, Turkey.

**Keywords:** Coronary Angiography/Adverse Effects, Arteriovenous Fistula, Endovascular Procedures

## Abstract

Arteriovenous fistula due to coronary angiography intervention is rarely seen. Arteriovenous fistulas may be asymptomatic according to the size of the shunt, as well as to the heart failure. In this case report, we aimed to share gradual transition from endovascular methods to surgery and why surgical treatment is required for a patient who developed arteriovenous fistula after coronary angiography.

**Table:** 

Abbreviations, acronyms & symbols		
**AVF**		**= Arteriovenous fistula**
**CAG**		**= Coronary angiography**
**DUS**		**= Doppler ultrasonography**

## INTRODUCTION

**Fig. 1:**
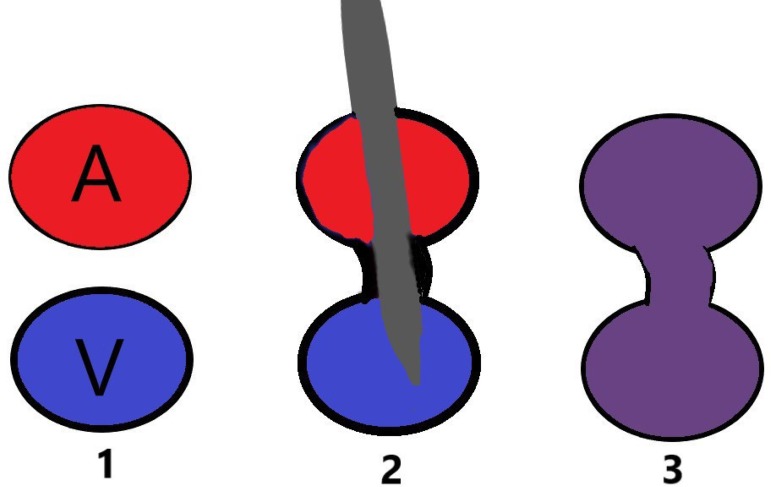
Arteriovenous fistula development.

Currently, coronary angiography (CAG) is the most effective method for diagnosis and treatment of coronary artery disease. Like every interventional procedure, some complications may arise even if the operator experience is sufficient during the CAG. Femoral arteriovenous fistula (AVF) rates varying between 0-0.08% have been reported in patients undergoing cardiac catheterization^[1,2]^. Arteriovenous fistula can be caused by simultaneous drilling of both arteries and veins by needle (Figure 1). Other adverse events after CAG include hematoma, pseudoaneurysm, dissection, embolism, infection, and extremity pain.

Murmur and stiffness can be detected on the intervention site on physical examination of AVFs. Diagnosis is mostly done with doppler ultrasonography (DUS) and angiography is used for definitive diagnosis.

Iatrogenic AVFs can be treated endovascularly and surgically. Endovascular methods have advantages such as early mobilization, short hospital stay and less risk of infection. If AVFs are not treated, they can cause cardiac insufficiency, edema and ischemia according to size and duration of shunt.

## CASE REPORT

A 59-year-old male patient had no complaints about AVF previously. His medical history includes hypertension, type 2 diabetes mellitus, 4 CAGs, and a coronary artery bypass graft surgery six years ago. In 2016, the latest history of CAG was available. During the examination for the fifth angiogram, DUS was performed on the leg with slight edema and murmur present on the previous CAG procedure site. An AVF was detected, between the right superficial femoral artery and superficial femoral vein with a diameter of about 3 mm in the DUS.

**Fig. 2:**
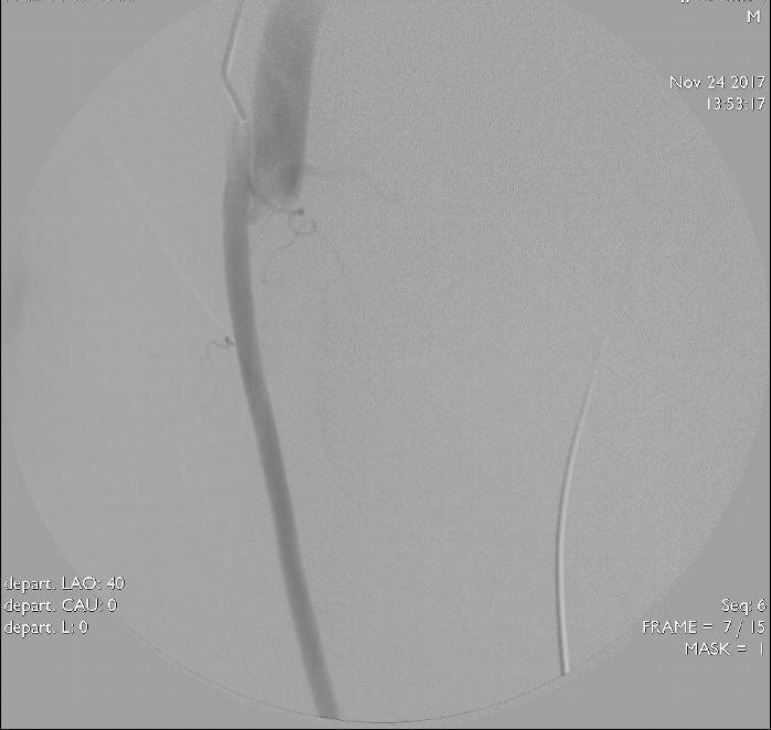
Angiographic image of fistula between superficial femoral artery and superficial femoral vein.

After consultation of interventional radiology, endovascular treatment was decided. Under local anesthesia, the right femoral artery was reached, and the right lower extremity angiograms were obtained after appropriate manipulations. A fistula was located between the superficial femoral arter and the superficial femoral vein (Figure 2). The femoral vein was reached after passing through the fistula tract. The catheter was then withdrawn slowly to try to embolize with cyanoacrylate (glue). However, the glue could not be stabilized due to the high flow. Although the balloon catheter was inflated for a long period with low pressure in the fistula region, the flow to the vein via fistula could not be prevented. Then, the patient was informed about the endovascular stent. However, the patient preferred a surgical intervention instead of stenting.

Common femoral artery, superficial femoral artery and superficial femoral vein were turned by right inguinal exploration with local anesthesia. An AVF of about 3 mm in diameter was seen 1 cm distal to the bifurcation. Clamps were placed on the arterial and venous sides and the fistula tract was cut from the center. Both vascular structures, first by the artery, were repaired by 6/0 prolene. The postoperative murmur disappeared and no fistula tract was seen in DUS.

## DISCUSSION

In parallel with the increasing number of CAG procedures in recent years, the number of complications has also increased. The number of puncture interventions, the posterior arterial wall penetration and the bigger size of punching needle increase the AVF formation after CAG^[3]^. It has been reported that properly performed vascular interventions, appropriate adjustment of anticoagulation and controlled blood pressure decrease the risk of fistula^[4]^.

In this case, the administration of large number of CAGs can be cited as the reason for the AVF. It was decided that an AVF of 3 mm in diameter should be treated even if asymptomatic, because progressive heart failure or limb ischemia might occur in late period. In the literature, it is stated that venous ulcer that doesn't heal, pigmentation, varicose enlargement due to prolonged AVF occurs and venous insufficiency and heart failure treatment are given to the patient^[5]^. 

Although there are no clear criteria in treatment approach, endovascular or surgical intervention should be considered for symptomatic patients, in cases of high-flowed heart failure and AVFs not self-closing within the first year^[6]^. It is also present in studies that one third of the iatrogenic AVFs spontaneously shut down within the first year^[2]^. 

In our study, we identified endovascular methods because of their advantages as the primary method for AVF closure. Because of the high flow on the fistula, the glue escaped to the venous system and prevented the AVF closure. Directly opening the fistula tract to the main vein prevented the application of large amount of glue. Treatment was terminated because venous thromboembolism could occur with excessive glue escaping to large veins with no valve structure. Other embolization materials have not been tested due to high flow.

An endovascular-coated stent, another treatment option, could be placed. Especially in the young patient group, we believe that stents in places with high mobility such as the inguinal region may be a thrombus source. It is also possible that covered stents placed close to the bifurcation points may close the side branches. In this case, the patient's opinion was also taken and primary vascular repair was performed with a skin incision of about 3 cm. As a surgical method, ligation, division and patch repair methods can be used according to the length of the fistula tract except for primary closure.

Surgical access of the lower and upper extremity arteries is easier than intrathoracic or intraabdominal arteries. We therefore believe that iatrogenic AVFs in the extremities need to be surgically treated instead of using coated stents. Surgical treatment is still the golden standard for these cases, as it is the definitive solution if endovascular procedures cannot be performed.

**Table:** 

**Authors’ roles & responsibilities**	
MI	Design of the work; or the acquisition, analysis, or interpretation of data for the work; drafting the work or revising it critically for important intellectual content; agreement to be accountable for all aspects of the work in ensuring that questions related to the accuracy or integrity of any part of the work are appropriately investigated and resolved; final approval of the version to be published
ÖT	Design of the work; or the acquisition, analysis, or interpretation of data for the work; drafting the work or revising it critically for important intellectual content; agreement to be accountable for all aspects of the work in ensuring that questions related to the accuracy or integrity of any part of the work are appropriately investigated and resolved; final approval of the version to be published
YD	Drafting the work or revising it critically for important intellectual content; agreement to be accountable for all aspects of the work in ensuring that questions related to the accuracy or integrity of any part of the work are appropriately investigated and resolved; final approval of the version to be published
VBT	Drafting the work or revising it critically for important intellectual content; final approval of the version to be published
ÖA	Drafting the work or revising it critically for important intellectual content; agreement to be accountable for all aspects of the work in ensuring that questions related to the accuracy or integrity of any part of the work are appropriately investigated and resolved; final approval of the version to be published
NG	Drafting the work or revising it critically for important intellectual content; final approval of the version to be published
